# Dynamical Motor Control Learned with Deep Deterministic Policy Gradient

**DOI:** 10.1155/2018/8535429

**Published:** 2018-01-31

**Authors:** Haibo Shi, Yaoru Sun, Jie Li

**Affiliations:** Laboratory of Cognition & Intelligent Computing, Department of Computer Science, Tongji University, Shanghai, China

## Abstract

Conventional models of motor control exploit the spatial representation of the controlled system to generate control commands. Typically, the control command is gained with the feedback state of a specific instant in time, which behaves like an optimal regulator or spatial filter to the feedback state. Yet, recent neuroscience studies found that the motor network may constitute an autonomous dynamical system and the temporal patterns of the control command can be contained in the dynamics of the motor network, that is, the dynamical system hypothesis (DSH). Inspired by these findings, here we propose a computational model that incorporates this neural mechanism, in which the control command could be unfolded from a dynamical controller whose initial state is specified with the task parameters. The model is trained in a trial-and-error manner in the framework of deep deterministic policy gradient (DDPG). The experimental results show that the dynamical controller successfully learns the control policy for arm reaching movements, while the analysis of the internal activities of the dynamical controller provides the computational evidence to the DSH of the neural coding in motor cortices.

## 1. Introduction

**Figure 1:**
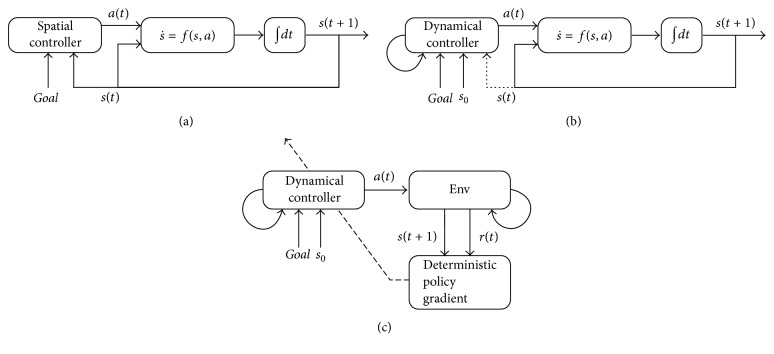
Schematic illustration of the dynamical control. (a) Conventional motor control takes the state feedback *s*(*t*) as input to generate the control signal *a*(*t*), and it behaves like a regulator or spatial filter to the feedback state. (b) The dynamical controller generates the control signal *a*(*t*) by its internal dynamics. Note that the dynamical controller loops by itself and theoretically the initial state *s*_0_ and the goal state are sufficient to generate the control command, with or without the feedback state (dotted arrow). (c) The dynamical controller is trained using DDPG with the reward information *r*(*t*) from the environment (shown as the Env box). The broken arrow indicates that the controller parameters are tuned with the gradients from DDPG.

Conventional computational studies of motor control adopt a spatial representation of the body's state, where the control command is generated by properly integrating the information of state feedback or state residual at a specific instant of time (Figure 1(a)) [1–3]. The parameters of the controller are adapted to capture the spatial patterns of the state input to generate the desired control command under a certain optimality principle [1, 4]. Recent neuroscience studies, however, have provided an alternative perspective, which emphasizes that the motor cortex serves as a dynamical system that generates the control command through its internal dynamics.

In recent neurophysiological studies aiming at deciphering the neural coding mechanisms in the motor cortex, Churchland and colleagues put forward a dynamical system hypothesis (DSH) to interpret the neural activities in the primary motor cortex (M1) during movement generation [5, 6]. Under this doctrine, the activities of the motor network are driven mainly by the interaction of the neural population within the network rather than responding to an external kinematic state. And the time-varying control command can be extracted from the population dynamics of the motor network [6, 7]. The motor cortex under the view of DSH can be described as a time-continuous dynamical system: $\dot{n}\left(t\right)=g\left(n\left(t\right)\right)+h\left(u\left(t\right)\right)$, where *n*(*t*) is the neural response during movement, $\dot{n}\left(t\right)$ is its derivative, and *u*(*t*) is the external input. Additionally, the motor network undergoes a preparatory period before movement execution, through which the network activity converges to a task-specific state. The converged state, in turn, is utilized as the initial state of the network in the movement execution period. The control command can be extracted sequentially when the network evolves under its own dynamics in the absence of the external input. With this arrangement, the computation of the control command can be embedded into a spatiotemporal transformation mechanism; that is, when the task-specific initial state is given, which is a spatial representation, the subsequent temporal evolution of the network and thus the output command can be unfolded deterministically.

Inspired by these findings, we proposed a computational model that exploited a dynamical controller to realize the spatiotemporal transformation mechanism. The dynamical controller was initialized with the task information and evolved under the constraint of its dynamics to elicit the desired control command (Figure 1(b)). Namely, the controller mapped the input task information to a temporal function of control commands. In the present study, the recurrent neural network (RNN) was adopted as the dynamical controller for its capability to approximate the dynamical system [8]. Accordingly, the learning of the control policy could be achieved by modifying the dynamics of the RNN controller, which was governed by the connectivity of the RNN.

Some previous related models have achieved the overall goal of mapping the motor task information to the control command sequence [9, 10]. Berniker and Kording verified the possibility of retrieving the command and state sequence from the task parameters using deep networks of the autoencoder architecture [9]. However, the computation that was achieved in their approach was essentially a spatiospatial transformation, where the task information was fed forwardly through the deep network and control commands for all time steps were output simultaneously, with each output dimension for a single time step. This method requires the control signal (for different time steps) to be accessible from different spatial locations, which is not biologically plausible. Also, previous computational models that incorporated the RNN dynamics for motor control captured the essential properties of motor circuits effectively [11, 12]. The multiphasic responses of the individual neurons and rotational patterns of the neuronal population were successfully reproduced. However, these models relied on the examples of optimal control policy given in advance as the training data to learn the controller. This is not plausible in biological control, which is learned in a trial-and-error manner; that is, no pregiven examples or immediate error is available for the synaptic adaptation.

To train the dynamical controller in the way of trial and error, we embedded it in the framework of reinforcement learning (RL) (Figure 1(c)). Among various RL methods, the policy gradient methods are suitable to learn the control policy in a continuous action and state space [13, 14]. Specifically, the deep deterministic policy gradient (DDPG) [15, 16] method was exploited in the present study to learn the control policy, because of the following two advantages. First, it does not require a probabilistic representation of the control policy and thus is compatible with the deterministic policy in motor control. Second, DDPG contains a term of the gradient of the action-value function with respect to the action, which is compatible with the learning method of the dynamical controller (detailed in the Methods and Materials).

The proposed model was evaluated in the task of arm reaching movement control. The results showed that the model successfully generated the accurate control commands and was able to reproduce the biomechanical properties and the neural responses found in the physiological experiments of arm reaching. Thus, this model could be a beneficial attempt to understand the neural substrate of motor control and for the engineering application in the field of robotic control.

The main contributions of this paper are as follows. First, a computational model that incorporates the neural mechanism of the spatiotemporal transformation from the task information to the motor commands was implemented, which provided a new paradigm for motor representation. Second, the proposed model did not rely on the pregiven sample data for training, but it learned the control policies through self-generated movements.

## 2. Methods and Materials

### 2.1. Deep Deterministic Policy Gradient

As the dynamical controller is embedded in the framework of DDPG, it is necessary to first introduce the DDPG method as a context. To define the terminology and symbols involved in DDPG, we first introduce the background of the Markov decision process (MDP).

Consider an MDP which consists of the state *s*_*t*_ from state space *𝒮*, the action *a*_*t*_ from action space *𝒜*, a stationary transition dynamics distribution *p*(*s*_*t*+1_∣*s*_*t*_, *a*_*t*_) that defines the dynamics of the environment, and a reward function *r* ∈ *𝒮* × *𝒜*. An agent optimizes a policy *μ*^*W*^ that is parameterized by *W* ∈ *ℝ*^*n*^ to maximize the cumulative rewards along the trajectory. At each time step, the state of the environment is updated with the action, and a reward *r*(*s*_*t*_, *a*_*t*_) is returned to the agent. The policy is improved upon the sequential information of state, action, and reward: {*s*_1_, *a*_1_, *r*_1_,…}. The return *ℛ*(*s*, *a*) at each time step is formulated as the discounted reward: *ℛ*(*s*, *a*) = ∑_*k*=*t*_^*∞*^*γ*^*k*−*t*^*r*(*s*_*k*_, *a*_*k*_), where *γ* is the discount factor and takes a value between 0 and 1. The action-value function *Q*^*μ*^(*s*, *a*) can be defined on the basis of the return function: *Q*^*μ*^(*s*_*t*_, *a*_*t*_) = *𝔼*[*ℛ*(*s*_*t*_, *a*_*t*_)∣*μ*^*W*^]. The objective function can also be defined as the action-value function with the initial state and action:

(1) $$\begin{array}{c} J\left(\;W\;\right)=Q^{\mu }\left(\;s_{1},a_{1}\;\right)=E\left[\;R_{1}\;|\;s_{1},a_{1},\mu^{W}\;\right]. \end{array}$$

Policy gradient (PG) methods directly adapt the parameters of the policy to maximize the objective function [13, 14], among which the deep deterministic policy gradient algorithm (DDPG) is developed for RL problems with continuous action and deterministic policy [15, 16]. It adopted an actor-critic architecture: the actor generates the control action and the critic estimates the value function of the policy. The actor and the critic are approximated with parameterized functions, *a*_*t*_ = *μ*(*s*_*t*_∣*W*^*μ*^) and *Q*_*t*_ = *Q*(*s*_*t*_, *a*_*t*_∣*W*^*Q*^), respectively, where *W*^*μ*^ and *W*^*Q*^ are the parameters for the actor and for the critic. Maximization over a continuous action space is achieved by altering the actor in the direction of the action gradient ∇_*a*_*Q*(*s*, *a*∣*W*^*Q*^). Accordingly, the deterministic policy gradient can be given as

(2) $$\begin{array}{c} \nabla_{W^{\mu }}J\left(\;W\;\right)=E\left[\;\nabla_{a}Q\left(\;s,a\;\right)\nabla_{W^{\mu }}\mu \left(\;s,a\;\right)\;\right], \end{array}$$

where ∇_*W*^*μ*^_*μ* is the gradient of the policy *μ* with respect to the actor parameter *W*^*μ*^.

**Figure 2:**
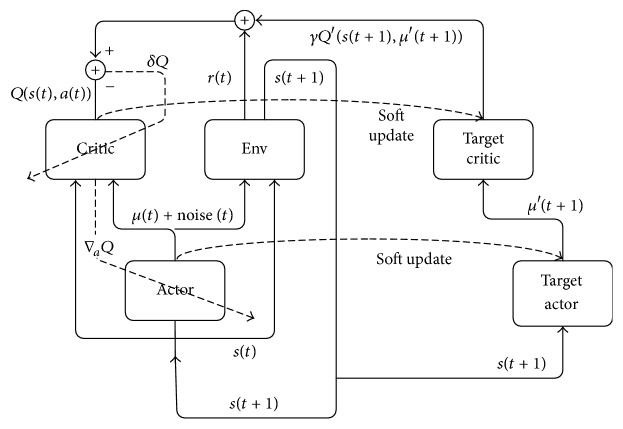
Schematic illustration of the deep deterministic policy gradient method. The critic network approximates the value function *Q*(*s*, *a*) by minimizing the TD error *δQ*. The actor network is updated with the gradient ∇_*a*_*Q* from the critic. Two sets of actors and critics are exploited for stability, shown as the boxes of “actor” and “critic” and the boxes of “target actor” and “target critic,” respectively. The target critic and target actor are updated by “soft update” for stability (arc dashed arrows).

As shown in Figure 2, the action-value function *Q* is updated with temporal difference (TD). The critic parameter *W*^*Q*^ is updated to minimize the TD error *δQ*, which is defined by the difference between the predicted *Q* value of the current time step and the target value:

(3) $$\begin{array}{c} \delta Q=r_{t}+\gamma Q^{\prime }\left(\;s_{t+1},\mu_{t+1}^{\prime }\;\right)-Q\left(\;s_{t},a_{t}\;\right). \end{array}$$

For learning stability, there are two sets of actors and critics. The additional set, which is denoted as target actor and target critic, is not a direct copy of the original set, but it slowly tracks the original set. In (3), the target value *r*_*t*_ + *γQ*′(*s*_*t*+1_, *μ*_*t*+1_′) is computed from the target networks. The update from the parameters of the original actor and critic networks *W*^*Q*,*μ*^ to the parameters of target networks *W*^*Q*′,*μ*′^ is determined by

(4) $$\begin{array}{c} W^{Q^{\prime },\mu^{\prime }}=\alpha W^{Q,\mu }+\left(\;1-\alpha \;\right)W^{Q^{\prime },\mu^{\prime }}, \end{array}$$

where *α* is a leaky factor with *α* ≪ 1 and defines how quickly *W*^*Q*′,*μ*′^ follows *W*^*Q*,*μ*^.

In this study, the instant reward *r* was comprised of several terms of penalties: *r*_*t*_ = −*δ*(*t* − *T*_*f*_) · (*s*^*∗*^ − *s*(*T*_*f*_))^*T*^*R*^*s*^(*s*^*∗*^ − *s*(*T*_*f*_)) − *a*_*t*_^*T*^*a*_*t*_, where *T*_*f*_ is the episode length, (*s*^*∗*^ − *s*(*T*_*f*_))^*T*^*R*^*s*^(*s*^*∗*^ − *s*(*T*_*f*_)) penalizes the error of the final state *s*(*T*_*f*_) from that target state *s*^*∗*^, and *a*_*t*_^*T*^*a*_*t*_ discourages the action amplitude through the trajectory. The components of state and action will be further described in the Arm Model section.

### 2.2. The Implementation of Actor and Critic

The critic was implemented with a multilayer perceptron (MLP), and the actor, which served as the dynamical controller in the proposed model, was implemented with a recurrent neural network (RNN). The critic network took the current state *s*_*t*_, target state *s*^*∗*^, and action *a*_*t*_ as inputs and approximated the action-value function *Q*(*s*_*t*_, *a*_*t*_) as output. The critic network had two hidden layers, each with 300 nodes. The state *s*_*t*_ and target state *s*^*∗*^ were fed to the input layer, while the action *a*_*t*_ was fed to the first hidden layer. Both hidden layers had Rectified Linear Units (ReLU) activation, which was defined as *f*(*x*) = max⁡(0, *x*), and the output units had linear activation.

**Figure 3:**
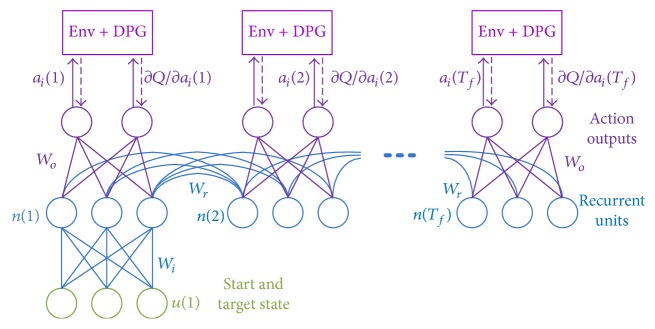
The HF learning of the RNN actor with the deterministic policy gradient. The RNN actor was unfolded in time to show the updates of the neural activity *n*(*t*), action *a*(*t*), and gradient propagation through time. The gradients of network weights *g*_*W*_ were acquired with gradients of the value function ∂*Q*/∂*a*(*t*) propagated from the critic in DDPG (see (6)). *W*_*i*_, *W*_*r*_, and *W*_*o*_ denoted the input, the recurrent, and the output weights, respectively. Note that only the task information (the start and target state) was fed to the network at the initial time step.

The RNN actor consisted of 100 interconnected units, which were fed with the concatenation of the start state *s*_0_, target state *s*^*∗*^, and the index of force field *C*, and outputted the action *a*_*t*_, that is, 6 muscle activation instances (detailed in the Arm Model section).  Figure 3 shows the connection of the actor network unfolded in time. The activity of the recurrent units *n*(*t*) had tanh activation:

(5) $$\begin{array}{c} n_{t}=\tanh\left(\;W_{i}\cdot {\left[\;s_{0},s^{*},C\;\right]}^{T}\delta \left(\;t\;\right)+W_{r}\cdot n_{t-1}\;\right), \end{array}$$

where *W*_*i*_ and *W*_*r*_ denote the input and recurrent weights, *δ*(*t*) ensures that the input was only applied in the first time step, and tanh activation was defined as tanh⁡(*x*) = (1 − exp⁡(−*x*))/(1 + exp⁡(−*x*)). To keep the muscle activation *a*_*t*_ positive, the output units had* sigmoid* activation: *a*(*t*) = *σ*(*W*_*o*_ · *n*(*t*)), where *W*_*o*_ is the weight of output projection, and the* sigmoid* function was defined as *σ*(*x*) = 1/(1 + exp⁡(−*x*)).

### 2.3. The HF Learning for the RNN Actor

The RNN actor was trained using Hessian free learning (HF). By incorporating the second-order information of the error surface during optimization, HF learning is effective in eliminating the “exploding and vanishing gradient problem” in training RNNs [17]. There are two main steps in HF learning: firstly, a Gauss-Newton matrix *G* is constructed from the outer product of the cost function with respect to the network parameters to represent the second-order information. Secondly, a conjugate gradient (CG) was conducted upon the Gauss-Newton matrix to optimize the network parameters.

To integrate the HF learning into this model, three main steps were taken. First, the gradients of the action-value *Q* with respect to muscle activation in each time step ∇_*a*(*t*)_*Q* were derived from the DDPG procedure. And the gradients were transmitted to the actor RNN through the output units (dashed arrows in Figure 3), which were back-propagated through time to the parameters of the network to get the gradient of *Q* with respect to the network parameters *g*_*W*_:

(6) ∂Q∂nt=∑k=tTf∂Q∂at∂at∂ntgW=∑t=0Tf∂Q∂nt∂nt∂W,

where *T*_*f*_ is the time horizon of the movement and *W* is the weights [*W*_*i*_, *W*_*r*_, *W*_*o*_] of the RNN actor. Second, the Gauss-Newton matrix *G* was constructed by the outer product of the parameter gradient: *G* = *g*_*W*_^*T*^*g*_*W*_. Third, the network parameters were optimized with CG iteration upon the Gauss-Newton matrix *G*. The Gauss-Newton matrix stayed the same within the loop of CG iteration, and it was updated for each batch of 64 reaches. To keep the stability of CG iteration and to regularize the optimized parameters, we incorporated Tikhonov regularization in this step. Specifically, the original Gauss-Newton matrix was added with a multiple of the identity matrix: *G* = *g*_*W*_^*T*^*g*_*W*_ + *λI*, where *λ* is the gain of the regularization and *I* is the identity matrix with the dimensionality of the parameters. And the CG iteration would be conducted on the new Gauss-Newton matrix.

### 2.4. Arm Model

The 2-link revolute model of the primate arm has been elaborately described in the literature. Here, we adopted the physics formulations given by [4]. The arm plant had two joints and was actuated by three pairs of muscles.

A detailed description of the muscular properties is given in the Appendix. The arm state $s=\left[\theta_{1}\theta_{2}\dot{\theta_{1}}\dot{\theta_{2}}\right]$ was represented by the joint angles and angular velocities, where *θ*_*i*_ is the *i*th joint angle and ${\dot{\theta }}_{i}$ is the angular velocity of the *i*th joint. The torques *τ*_*i*_ on each joint could be calculated by a nonlinear function of the muscle activation and the muscle state (see the Appendix). Driven by the joint torque *τ*, the angular acceleration in the joint space was subject to the following equation:

(7) $$\begin{array}{c} M\left(\;\theta \;\right)\ddot{\theta }+C\left(\;\theta ,\dot{\theta }\;\right)+B\dot{\theta }=\tau , \end{array}$$

where *M*(*θ*) is the inertia matrix and depended on the joint angles, $\ddot{\theta }$ is the angular acceleration, $C\left(\theta ,\dot{\theta }\right)$ is a vector defining the centripetal and Coriolis forces depending on both the joint angles and the joint angular velocities, *B* is the joint friction matrix with respect to the angular velocities, and *τ* is the joint torque. The force field could be applied by manipulating the matrix *B*: *B* = [0.05,0; 0,0.05] for no force field, [0,0.05; −0.05,0] for clockwise force field, and [0, −0.05; 0.05,0] for anticlockwise force field.

## 3. Results

We examined the capability of the proposed model to generate the optimal control policy for planar arm reaching. The RNN actor generated muscle activity *a*_*t*_ as the control command to drive the limb model. Each reaching movement had a fixed time horizon *T*_*f*_ = 25; namely, all episodes had the same length of 25 time steps. For each time step, the plant state *s*_*t*_, the action *a*_*t*_, the new state *s*_*t*+1_, and the network activities were stored to a data buffer with a buffer size of 10^5^. The discounted factor *γ* was kept at 0.99. To avoid the correlations between samples, the critic network and the target critic network were trained using the scrambled samples from the data buffer. The critic networks and the RNN actor network were updated for every minibatch, which was a buffer of 64 episodes. The gain of the regularization to the network weights *λ* was set to 0.03. The regularization term was not trivial, since it could prevent the output torque and network activity from dramatically changing. Without the regularization, the reaching trajectories would become heavily curled.

**Figure 4:**
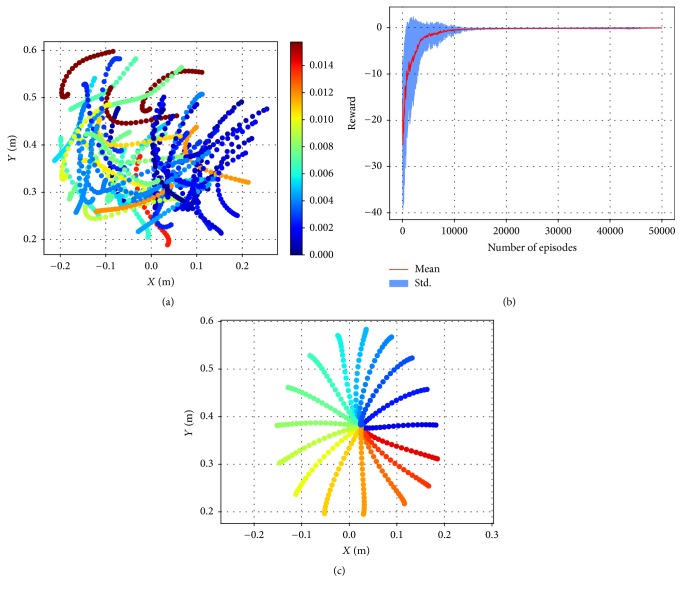
Training and validation of the reaching movement generation. (a) The trajectories of random reaches after training. The start and target points were drawn from a disk-shaped work space. The trajectories were color-coded by the scale of error that measured the distance between the end state and the target state. (b) The cumulative reward versus the number of episodes. (c) The center-out reaching trajectories generated by the trained model.

The model was first trained for randomly generated reaches. The start and end points of each reach were confined in the work space of a circle centered at  [0.0265, 0.3833]  and with a radius of 20 cm. The start and end points were described by the Euclidean coordinates of the arm's end-effector. As the arm state was represented in the joint space, we first transformed the target position from the Euclidean space to the joint space. The transformation was achieved by iterating the randomly initialized joint state along the direction where the Euclidean error of the current Euclidean state was minimized. For each trial of movement, a pair of start and target points was drawn randomly from the work space. The uniform distribution of the start and end points excludes the bias that may be introduced by the preference of the task parameters. The reaching trajectories of the randomly sampled points are illustrated in Figure 4(a). The cumulative reward versus the number of episodes was plotted in Figure 4(b). The red curve shows the mean cumulative reward over 15 randomly initialized models, and the area of the mean ± standard deviation is shaded in blue. The training was validated by the center-out reaching task (Figure 4(c)). In the center-out task, the reaches always started from the center and aimed at a surrounding point with a fixed distance of 18 cm. The surrounding target points were arranged evenly along a circle with an angular interval of *π*/8.

**Figure 5:**
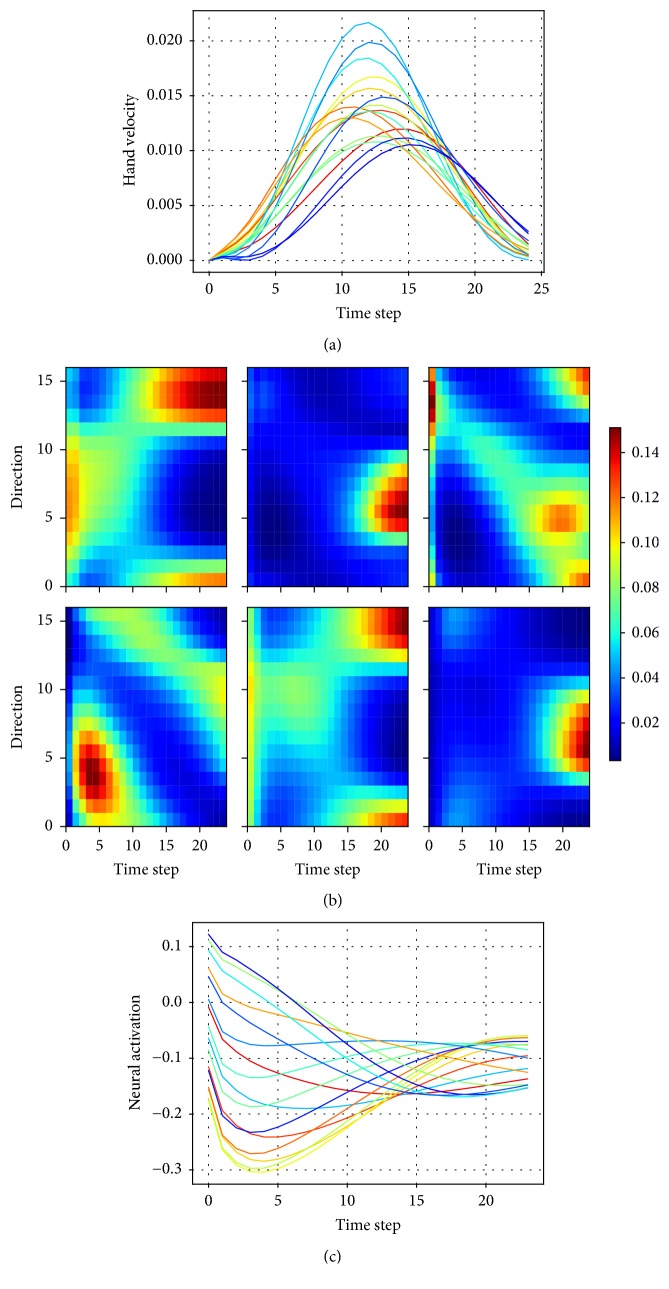
Training and validation of the reaching movement generation. (a) Velocity profiles of reaching movements in different directions. The plot of hand velocity versus time of all the center-out movements displayed a bell-shaped profile. Color coding was as in Figure 4(c). (b) The activation of six muscles for 16 reaching directions (each panel for a muscle). Horizontal and vertical axes represent time and reaching direction, respectively. (c) Activation of a typical neuron in the dynamical controller for movements in different directions.

The model successfully generated accurate control commands to drive the arm for center-out reaches after training, and it captured some essential biomechanical and neural properties resembling the real arm reaching movements. Specifically, the trained model elicited relatively straight hand trajectories and bell-shaped velocity profiles (Figures 4(c) and 5(a)). The circled lines in Figure 4(c) depict the trajectories generated by the controller. Circles were plotted with the same time interval along each trajectory, and the density of the dots reflected the hand velocity. For clarity, the velocity profiles of the same set of movements were also plotted. The hand velocities for all movements displayed a bell-shaped profile and were insensitive to the movement amplitude and direction. Figures 5(b) and 5(c) show the muscle activity and the responses of an individual neuron in the controller network during movement for reaches of 16 different directions. The muscle activities and neural responses displayed a smooth change across different directions of reaching movements. These features were in accordance with findings of the biomechanical experiments in the literature [2]. Considering that the control commands could be identified with the task information, the continuity of the control commands across different movements indicated the continuity in the task information.

**Figure 6:**
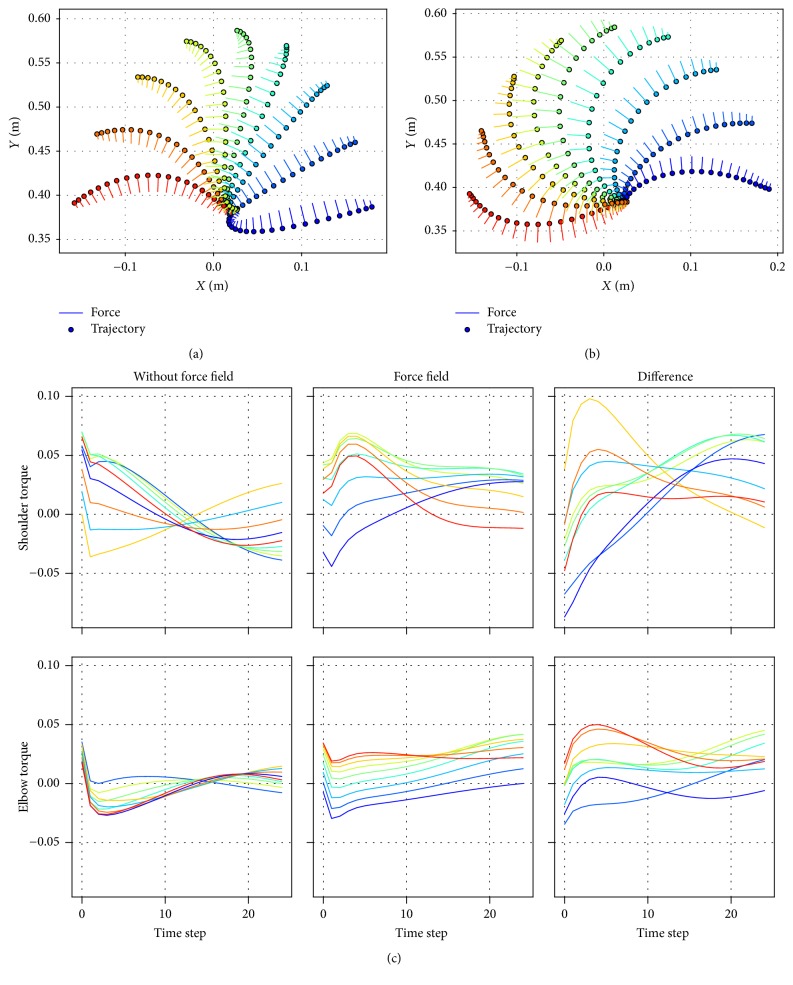
Curled trajectories under force field. (a) The reaching trajectories adapted for anticlockwise force field. (b) The reaching trajectories adapted for clockwise force field. Trajectories in both (a) and (b) curved to the opposite direction of the perturbing force. The short lines attached to each circle visualize the direction and the strength of the deflection force. (c) The difference between torques without force field and under force field. The torques for the two joints are arranged in rows. The torque difference demonstrates the overcompensation at the early phase of the movements.

Adaptation of the controller under a force field yielded curled trajectories. The force field could be applied by manipulating the matrix *B* in (7) of the arm model. The diagonal elements of the matrix *B* related the velocity state and the friction: friction torque on each joint was proportionate to the angular velocity of the same joint. And the off-diagonal elements defined the strength of the curling force field: the velocity of one joint angle would cause the skewing torque on the other. The matrix *B* was assigned with [0.05,0; 0,0.05] for the null field, [0,0.05; −0.05,0] for clockwise force field, and [0, −0.05; 0.05,0] for anticlockwise force field. The direction of the deviating force resulting from the angular velocity was perpendicular to the direction of the hand velocity. The adapted trajectories showed an opposite direction of deflection against the direction of applied force (Figures 6(a) and 6(b)). For example, in Figure 6(b), the overall direction of the force field (shown as the dashes attached to the circles of the trajectories) went clockwise, and the trajectories curved anticlockwise. This could be attributed to the process of reoptimization under the force field. In the theory of reoptimization, the optimal command overcompensated the perturbing force at the early stage of movements [18, 19]. This could be verified in Figure 6(c), where the difference between the joint torques in the force field *τ*_force_field_ and the torques without the force field *τ* had larger amplitudes at the early stage of movements.

**Figure 7:**
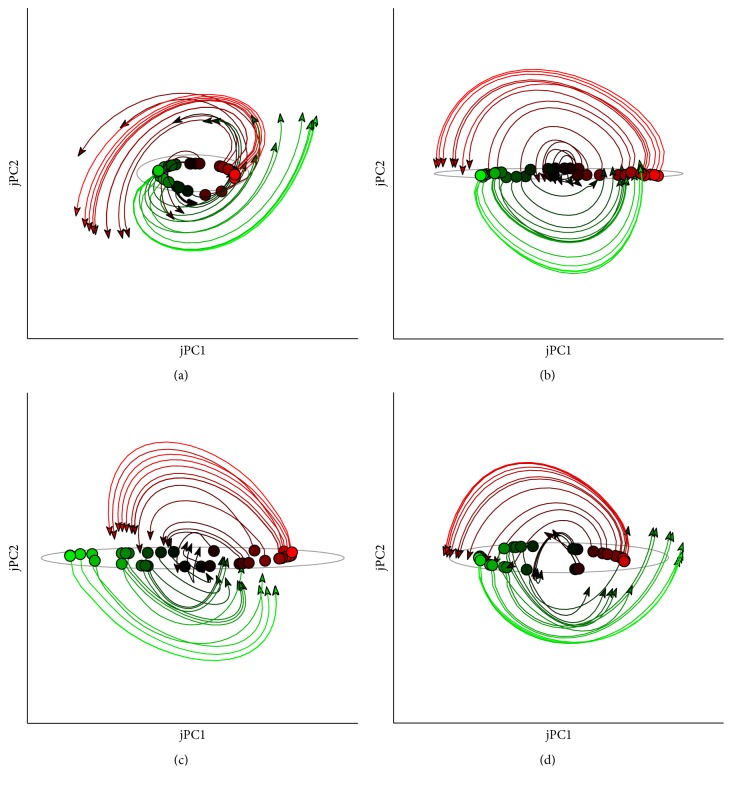
Rotational trajectories of population activities in the dynamical controller. The jPCA analysis was performed on the data from four trained controllers. Neural activities during the movement were projected to the first pair of jPCs. The projected trajectories for movements of different directions showed consistent rotations.

The population dynamics of neurons in the dynamical controller during motor generation were also analyzed and compared with the findings from physiological studies of DSH. The neural activities were analyzed with a newly developed method, jPCA analysis [5], which had found the consistent rotational structure in the population activities (Figure 7). The jPCA analysis is developed to verify the DSH by extracting the rotational transformation underlying the aggregate neural states [5, 6]. The computation behind this method is to fit the derivative of neural states $\dot{n}\left(t\right)$ with a product of the transition matrix and the neural states $\dot{n}\left(t\right)=M_{\text{skew}}\cdot n\left(t\right)$, and the transition matrix is constrained to be skew-symmetric (*M*_skew_ = −*M*_skew_^*T*^). The dominant rotations are those corresponding to the first few pairs of the eigenvalues of the fitted skew matrix, which have imagery values and come in pairs. The rotational trajectories could be obtained by projecting the population activities onto the eigenvector of the dominant eigenvalues.

The results showed that there were coherent rotational transformations for the neural states in the jPC space (projection on the first pair of the eigenvectors of the fitted skew matrix). The neural states underwent rotation with the same frequency, and the different target states *s*^*∗*^ were related to the different initial phases and amplitudes. Take the neural activities in Figure 7(a) as an example; the first pair of the eigenvalues were [0.138*i*, −0.138*i*], and the dominant frequency was 0.022/time_step. The dominant rotation accounted for a major part of the variance of the total neural activities (52% on average for all trials). This was in accordance with the findings from neurophysiological studies [5, 6], in which the quasi-oscillations were the most prominent features in the neural responses during reaching in M1 circuitry. The jPCA was applied to quantitatively relate the quasi-oscillation to the sparse rotational structures, thus attributing it to the neural dynamics of the M1 network. The common presence of rotational dynamics in both the dynamical controller and its biological counterpart (M1 circuitry) suggested that the present study can also provide computational evidence to the DSH of motor processing in the motor cortex.

## 4. Discussion

This study implemented a computational model of motor control based on the DSH of neural coding mechanism in motor cortex. The motor generation was achieved through a spatiotemporal transformation mechanism with a dynamical controller. The control commands could be unfolded from the dynamics of the controller network when its initial state was specified with the task information. Motor learning was implemented with the DDPG framework. And the two processes were integrated by embedding the dynamical controller in the DDPG as the actor network, which allowed the model to be trained in a trial-and-error manner. As shown from the experiments of arm reaching movement control, the model successfully generated an accurate control policy that captured the key properties found in the biomechanic experiments. And the analysis of the internal dynamics of the controller provided the computational evidence for the dynamical system hypothesis of the neural coding in motor cortices during movement generation.

The model proposed in this study has a loose resemblance to the specializations of the biological nervous system, where the DDPG learning corresponds to the rewarding circuitry [20, 21], and the RNN actor corresponds to the cortical motor areas. It has been revealed that the nigrostriatal dopamine neurons in the basal ganglia represent the temporal difference error (TD error) [22], as the learning of the critic in RL. These different modules interact with each other to fulfill the cognitive tasks of motor control and motor learning. In our model, this interaction between the learning modules can be recognized as the propagation of the DPG gradients ∂*Q*(*s*, *a*)/∂*a*, which are transmitted from the critic network to the output units of the RNN actor at each time step for the modification of the actor parameters *W*^*μ*^ (Figure 3). The supervised learning of the RNN controller and the reinforcement learning of DDPG are thus integrated in the same framework.

The generation of time-varying control commands from an initial state in the dynamical state can be regarded as a spatiotemporal transformation. This mechanism can be applied in the hierarchical control in future studies. With the spatiotemporal transformation as the low-level controller, the higher-level control module only needs to send out the motor goal in terms of spatial information, leaving the computation of the time-varying control commands to the low-level control. Under this hierarchical architecture, a complex movement can be implemented by sequentially initializing the low-level controller.

## Figures and Tables

**Figure 8:**
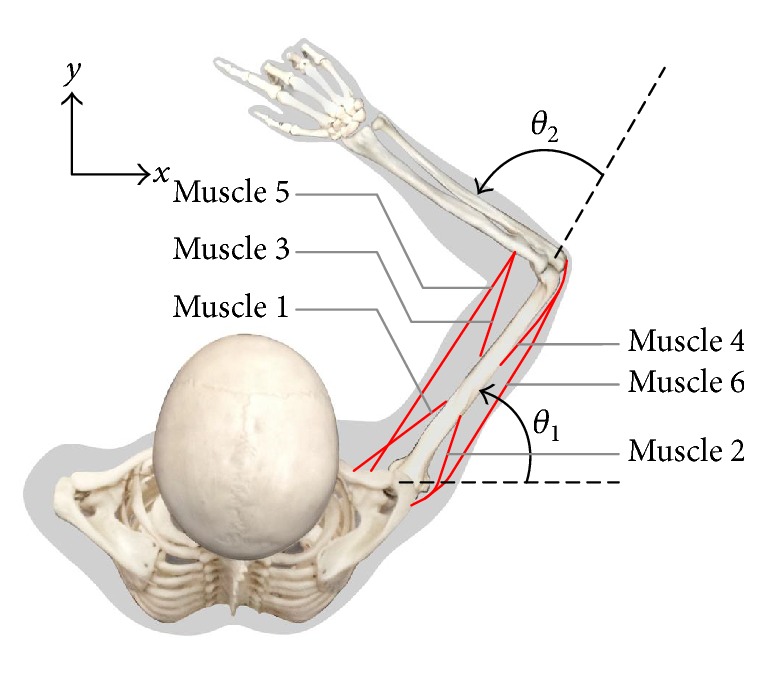
Musculoskeletal model of the arm.

## Appendix

As shown in Figure 8, the arm plant is driven by three pairs of muscles. One pair consists of biarticulate muscles (from shoulder to forearm), while the other two, respectively, actuate the shoulder or elbow joint (monoarticular shoulder flexors, monoarticular shoulder extensors, monoarticular elbow flexors, monoarticular elbow extensors, biarticular flexors, and biarticular extensors). Each pair of muscles is a pair of flexor and extensor. The activation is provided by the network output *a*(*t*). Given the muscle activation, the force exerted is also nonlinearly dependent on muscle length *l* and contraction speed *v*:

(A.1) $$\begin{array}{c} \text{Force}\left(\;t\;\right)=a\left(\;t\;\right)\cdot f_{l}\left(\;l\left(\;t\;\right)\;\right)\cdot f_{v}\left(\;l\left(\;t\;\right),v\left(\;t\;\right)\;\right). \end{array}$$

With the muscle tension and the anatomical configuration, which is reflected by the moment arm matrix *L*, the joint torque *τ* can be calculated by

(A.2) $$\begin{array}{c} \tau \left(\;t\;\right)=L\cdot \text{Force}\left(\;t\;\right). \end{array}$$

The moment arm matrix describes the relationship between joint torques and muscle forces under certain gesture. The trivial variations of the moment arms due to the change of joint angle are ignored for simplification. The moment arm matrix is given as

(A.3) $$\begin{array}{c} L=\left[\begin{array}{cccccc} 2 & -2 & 0 & 0 & 1.5 & -2\\ 0 & 0 & 2 & -2 & 2 & -1.5 \end{array}\right]. \end{array}$$

The columns of *L* correspond to six muscles. The muscle length is fitted using the function of current deviance from the optimal joint angle *θ*^0^ and the optimal length *L*^0^. The matrix *θ*^0^ of size 2 × 6 indicates the optimal angle of the two joints for each of the six muscle groups. Similarly, six columns in *L*^0^ indicate the optimal length for each of the six muscles. Same as in the matrix *L*, zero-value elements in *θ*^0^ and *L*^0^ represent the anatomical absence of the corresponding muscles. For the *i*th muscle group, the dependence of current length on the deviance is given as follows:

(A.4) li=1+T1,i·θ01,i−θ1L0i+T2,i·θ02,i−θ2L0iθ0=2π36015.05.02003.92.120080.86109.3292.9691.52L0=7.323.266.43.265.954.06.

The derivative of muscle length ${\dot{l}}_{i}$, that is, the muscle contraction velocity, can be achieved using a weighted summation of the joint angle velocity ${\dot{\theta }}_{i}$, which is also parameterized by the moment arm matrix *L* and the optimal length *L*^0^:

(A.5) $$\begin{array}{c} {\dot{l}}_{i}=\frac{T_{1,i}\cdot \dot{\theta_{1}}}{{L^{0}}_{i}}+\frac{T_{2,i}\cdot \dot{\theta_{2}}}{{L^{0}}_{i}}. \end{array}$$

The muscle length and velocity are normalized by dividing *L*^0^ and thus they can be seen as relative length and velocity. The nonlinearity terms *f*_*l*_(*l*) and $f_{v}\left(l\left(t\right),\dot{l}\left(t\right)\right)$ implement the scaling effect of the fascicle force-length relationship and force-velocity relationship. The *f*_*l*_(*l*) function and the $f_{v}\left(l\left(t\right),\dot{l}\left(t\right)\right)$ function are approximated as follows:

(A.6) fll=exp⁡−liφ−1ωρ⁡fvli,l˙i=Vmax⁡−l˙iVmax⁡+cV0+cV1lil˙il˙i≤0bV−aV0+aV1li+aV2li2l˙ibV+l˙il˙i>0,

where *φ* = −1.55, *ω* = 0.81, *ρ* = 2.12, *V*_max⁡_ = −7.39, *c*_*V*0_ = −3.21, *c*_*V*1_ = 4.17, *b*_*V*_ = 0.62, *a*_*V*0_ = −3.12, *a*_*V*1_ = 4.21, and *a*_*V*2_ = −2.67.
